# How Shall We Best Insert a Gold Filling?

**Published:** 1898-08

**Authors:** Arthur Galusha Smith

**Affiliations:** Peoria, Ill., in Illinois Society.


ARTICLE III.

### HOW SHALL WE BEST INSERT A GOLD FILLING?

DR. ARTHUR GALUSHA SMITH, PEORIA, ILL., IN ILLINOIS SOCIETY.

It has required years to bring the gold filling to its present degree of usefullness and perfection, but its history, though interesting, does not concern us. What we should strive to find out and thoroughly understand is, what constitutes, to-day, the best gold filling, and what is, all things considered, the best means of producing it.

I need only allude to the two distinct kinds of gold used in fillings, namely, "soft" and "cohesive." A "soft" foil, strictly speaking, cannot be rendered in any degree cohesive by annealing. However, many of the so-called soft foils now on the market will develop a certain amount of cohesion when annealed. A "cohesive" foil on the contrary is rendered so sticky by annealing that a mere touch will unite two pieces, while a blow or heavy pressure will make them become as if of one piece. These differing properties of the gold as it comes to our hands from the dealers, are fixed and unchanging and must be carefully borne in mind, as they make the two kinds of gold almost exact opposites in working properties.

However perfect our preparation, however neat and beautiful the finish of the filling, if at any point we fail materially in our adaptation, much of our work is sure to fail, and when we see it as the years go by, we ourselves know that we have failed and fallen short of what we should have accomplished; even though our patients are satisfied that it was "all their fault," and cheerfully have the work replaced with faith in us unshaken and unwavering.

The teeth are by nature designed to withstand pressure, but not blows.

Dr. Black, two or three years ago, showed us conclusively that it was almost impossible for a person to injure the teeth by exerting the extreme force of the muscles of mastication, provided that force be exerted gradually; but how different is the result of the same muscular action when we bite suddenly on some hard substance and thus give a blow to the teeth.

In other words, we recognize the necessity for avoiding blows on the teeth and instruct our patients to void them as far as possible, and yet we ourselves make such large use of them in condensing gold. Is not this an illogical position?

I have seen many large and beautiful fillings that were made with the mallet, but in many of them there have developed large pits or soft places, showing the condensation was not even. Again, many of the teeth containing large malleted fillings are so badly checked as to be beyond repair as soon as the first chip falls out of the cavity margin.

Loosening and soreness of the teeth is always the immediate result of any considerable malleting, and this sometimes never entirely subsides.

With the use of hand pressure all these disagreeable sequele are much lessened or entirely done away with. How many times after the insertion of a large gold filling have my patients remarked in surprise "Why, doctor, that tooth wasn't even sore the next day."

Finally we have to consider the operation from the patient's standpoint, and here I can speak with feeling and appreciation and utterly free from bias, for I think that I have had gold fillings put in by almost every known process except that of the Herbst burnishers, and from the patient's standpoint I must say that no method of condensing gold with which I am acquainted is so free from discomfort and objectionable features as hand pressure.

There is no noise or jar of any kind connected with the operation, and this fact makes the nervous strain on the patient much less, and this attitude of ease and rest in the person on whom we are working adds much to the rapidity and freedom from tension with which we do our work.

My patients are warm in their praises of hand pressure, and it was largely this fact that induced me to persist in using it and finally rely on it altogether. When they have a filling done in my office for the first time, they almost invariably express the greatest relief and astonishment at the absence of what they usually term the "pounding" or "that little thumping machine." At this time, when we are ostensibly striving so earnestly to take away those things which are sources of discomfort to our patients, can we afford to overlook anything which is so simple, so effective and so free from discomfort?

No one mallets a filling from the very start. A variable amount of hand pressure is necessary to carry the first few pieces of gold to place, whatever method we use afterward.

If this is more effective in carrying gold to place against the walls of the cavity at the start, can there be any reason why it will not secure a better adaptation throughout the entire operation?

I fully realize that no two men work alike, and that a method which is a success in the hands of one operator will be much less satisfactory in the hands of another; but I do believe that, with all of us, this hand pressure concondensation is worthy of an earnest trial.

I formerly used the mallet as exclusively as any man possibly could, and considered everything else too utterly out of the question, to be even seriously considered, but I came in close personal contact with a most expert operator who was an enthusiastic advocate of hand pressure. The beautiful results which he achieved, and the ease with which he worked in cavities which were difficult of access to me and my mallet, set me to thinking and finally to experimenting with that which I had formerly been content with ridiculing. It required considerable time for me to get the muscles of my fingers trained so that they could stand the long continued exertion, and I had better pause here before I finish, and say that it has not been a question of the easiest way to do a gold filling that I have been considering, but this was the only difficulty encountered, and it has long since disappeared.

Some day, when you have a cavity that is difficult to reach in a satisfactory manner with your mallet—and you won't have to wait long for this opportunity—and rather small, just try hand pressure for awhile. If you get tired, take up your mallet and finish out with that, but keep on trying till your muscles become thoroughly accustomed to the work, and it will not be till then that you can pass a fair judgment on the method, and I am sure that you will do with your mallets just what I did with mine—let them lie in the drawer unused for a year or two, and finally discard them altogether.—*Dental Brief.*

